# Predictors of mortality among critically ill SARS-CoV-2 infected patients—a retrospective cohort study, Kerala, India

**DOI:** 10.3389/fpubh.2025.1635476

**Published:** 2025-08-28

**Authors:** Anil Sathyadas, Aravind Reghukumar, Thekkumkara Surendran Anish, Gnanaseelan Kanakamma Libu, Vishnu Narayan Kiran, Kannamkottapilly Chandrasekharan Prajitha, Anisha Sharahudeen, Dhanusha Chandran, Muralidharan Rohini Athirarani, L. Sindhu, Parackalparambil Sukumaran Sona, Thekkumkara Prabhakaran Sreekanth, Sujatha Chintha, Thomas Iype, Muthezhathu Kesavadas Suresh, Kavitha Ravi, K. Rajamohanan, Thomas Mathew, John Panicker, M. K. C. Nair, A. Nizarudeen, Parameswaran Hari

**Affiliations:** ^1^Dr MRS Menon Foundation, Government Medical College, Thiruvananthapuram, India; ^2^Department of Emergency Medicine and Critical Care, Government Medical College, Thiruvananthapuram, India; ^3^Department of Infectious Diseases, Government Medical College, Thiruvananthapuram, India; ^4^Kerala One Health Centre for Nipah Research and Resilience, Kozhikode, India; ^5^Department of Community Medicine, Government Medical College, Kerala, India; ^6^Department of Pulmonary Medicine, Government Medical College, Thiruvananthapuram, Kerala, India; ^7^National Institute of Epidemiology, ICMR, Chennai, India; ^8^Department of Community Medicine, Government Medical College, Thiruvananthapuram, India; ^9^Government College of Nursing, Thiruvananthapuram, India; ^10^Government College of Nursing, Kollam, India; ^11^Kerala Nurses and Midwives Council, Thiruvananthapuram, India; ^12^Department of Neurology, Government Medical College, Thiruvananthapuram, India; ^13^Department of Internal Medicine, Government Medical College, Thiruvananthapuram, India; ^14^Department of Pathology, Government Medical College, Thiruvananthapuram, India; ^15^School of Public Health, Kerala University of Health Sciences, Thiruvananthapuram, India; ^16^Ex-medical Superintendent, Government Medical College, Thiruvananthapuram, India; ^17^Medical College of Wisconsin, Milwaukee, WI, United States

**Keywords:** critically ill COVID-19, COVID-19 mortality, mortality predictors, Kerala, India

## Abstract

**Background:**

Preventing the in-hospital mortality of critically ill patient is the last opportunity to saves lives during a pandemic. It was a need for the hospital settings of global south to further prioritize the individuals in this vulnerable group to allocate scares resources because of large numbers of such patients admitted in hospitals during pandemics. We, in this study flag the risk factors for in-hospital mortality for critically ill patients at the time of a pandemic like COVID-19.

**Methods:**

This retrospective cohort study aimed to analyze the in-hospital mortality rate and predictors of mortality of patients with critically ill SARS-CoV-2 infection admitted to a Level 3 multi-disciplinary intensive care unit in India from15th September 2020 to 31st March 2021. We compared the incidence proportion of in-hospital mortality in different subgroups. We calculated the relative risk (RR) of clinical and biochemical factors under study for mortality outcome. We used principal component analysis to identify risky groups because the mortality predictors were found to have been highly correlated with one another in univariable analyses.

**Findings:**

Of the 431 adult study participants with a median (IQR) age of 48 (34–60) years, 26.2% (*n* = 113) were aged 60 years or above, and 58.9% (*n* = 254) were men. Significant predictors of mortality in patients with severe SARS-CoV-2 infection were; age more than 60 years [RR 1.67 (1.36–2.02), *p* < 0.001], chronic kidney disease [RR 1.7 (1.01–3.14), *p* = 0.022], systemic arterial hypertension [RR 1.69 (1.32–2.15), *p* < 0.001], diabetes mellitus [RR 1.22 (1.00–1.49), *p* = 0.042], coronary artery disease [RR 1.59 (1.03–2.43), *p* = 0.012], any malignancy [RR 2.79 (1.17–6.65), *p* = 0.020], SARS-CoV-2 unvaccinated status [RR 1.59 (1.33–2.22), *p* = 0.008] COVID ARDS [RR 5.34 (2.54–11.25), *p* < 0.001], COVID Bronchopneumonia [RR 1.16 (1.03–1.31) *p* = 0.017], sepsis [RR 4.28 (1.76–10.38) *p* = 0.001], septic shock [RR 25.65 (3.48–189) *p* = 0.002], acute kidney injury [RR 10.59 (3.25–34.45) *p* < 0.001] and infection-related ventilator-associated condition (IVAC) [RR 2.13 (1.43–3.17) *p* < 0.001].

**Interpretation:**

Renal insufficiency, transaminitis, coronary artery disease and elevated inflammatory markers, comorbidities and lack of vaccination, Pneumonia, Breathlessness and ARDS, sepsis and septic shock, cough, and diarrhea at the time of admission were identified as nine domains/variables that contributed to mortality. It is relevant in the clinical setting of LMICs (low- and middle-income countries) with limited healthcare resources. These predictors would help in prognostication of the disease and guide in rationalizing the management of patients in the context of pandemic threats.

## Introduction

The case fatality rate (CFR) of COVID-19 is lower than that of many pandemics in the past because of public health interventions including prompt and extensive vaccinations (1). However, the risk of dying following SARS-CoV-2 infection varied between countries and communities. The direct and indirect risk of mortality due to the pandemic was higher in countries of global south (2). Identifying the clinical, biochemical, and epidemiologic risk factors for mortality among patients with severe SARS-CoV-2 infection in a resource-constrained LMIC (Low and Middle Income Countries) setting helps in triaging high-risk patients, delivering focused critical care interventions and prioritizing limited resources.

A recurring pattern noticed with every pandemic, is that the mortality burden tends to concentrate in regions with weak health systems (3). In India, the early waves of the COVID-19 pandemic imposed significant mortality challenges, directly through infections and indirectly through collateral damages (2, 3). However, mortality rates vary across communities, largely influenced by local healthcare systems’ response time, effectiveness and resilience. Kerala, among the Indian states, witnessed comparatively lower mortality rates which could be attributed to evidence-based pandemic management strategies (4–6). Notably, the state implemented a protocol-based clinical management of SARS-CoV-2 infection, integrating medical care into universal health coverage and providing free treatment through public health facilities (7). Patients were categorized based on clinical severity with the highest grade (Category C) managed in dedicated COVID hospitals featuring specialized Intensive care units (ICU) and multi-disciplinary intensive care units (MDICU). Understanding the risk of death and the associated factors of death in the clinical setting of an ICU located in an LMIC is critical to curbing the impact of pandemics. This study examined the in-hospital mortality rate and associated risk factors among severe COVID-19 patients admitted to the MDICU at Government Medical College Thiruvananthapuram (GMCT), Kerala, India.

## Materials and methods

We conducted this retrospective cohort study among critically ill COVID-19-positive patients admitted to the MDICU of GMCT, one of the largest COVID hospitals in Kerala, India. GMCT was the apex center for all public sector hospitals managing severe COVID-19 in south Kerala. All the consecutive critically ill patients above 12 years of age with SARS-CoV-2 admitted to MDICU from 15th September 2020 to 31st March 2021 were included in the study. After obtaining ethics committee clearance for the study, the hospital records were assessed to collect clinical information at the time of admission to the MDICU, the development of complications if any, and the outcome of the patients (in-hospital mortality or discharge).

We recorded clinical, biochemical, and radiological data in a structured proforma. The major outcome variable was the in-hospital mortality and the effects of sociodemographic variables, vaccination status, comorbidities, and clinicopathological features on the outcome were evaluated. The biochemical and clinical indices captured included total white cell count, neutrophil count, lymphocyte count, platelet count, Erythrocyte Sedimentation Rate (ESR), C-reactive protein (CRP), D-dimer, albumin, procalcitonin, ferritin, lactate dehydrogenase (LDH), creatine phosphokinase (CPK), troponin-T (Trop-T), arterial blood gas (ABG), interleukin-6 levels (IL-6), liver function tests (LFT), renal function tests (RFT), serum electrolytes, blood culture and susceptibility, sputum/Endotracheal (ET) aspirate culture and susceptibility, PT-INR, aPTT, neutrophil-lymphocyte ratio, SOFA score and APACHE SCORE. All patient records with the outcome status were included in our analysis. Patients or caregivers were approached over the phone to get information on the outcome if the data on the case sheet was ambiguous.

## Data analysis

We coded and analyzed the data using IBM SPSS Statistics for Windows, Version 27.0 (Armonk, NY: IBM Corp.). We conducted a descriptive analysis of the study participants. We evaluated the proportion of in-hospital mortality and estimated the relative risk of all potential clinical and biochemical factors for mortality, along with their 95% confidence limits. We expressed the dose–response relationship between hierarchical exposures and the chance of mortality as a change in odds ratios. The Chi-square test was used to detect the statistical association between different variables and mortality and the chi-square for the linear trend was used to test the dose–response relationships. We did not use the conventional regression techniques such as multivariable analysis because the exposure factors were found to be highly correlated with each other. Rather we tried to find out the combinations of clinical, immunological, and biochemical combinations that lead to mortality in severe COVID disease. Factor reduction technique (principal component analysis) was used to identify the combinations that lead to the outcome variable, in-hospital mortality. Moreover, our data showed that input variables are correlated and are suited for factor analysis (Kaiser-Meyer-Olkin (KMO) measure of sampling adequacy = 0.541 and *p* < 0.001 for Bartlett’s test for sphericity).

## Results

The study included 431 adult SARS-CoV2-infected critically ill patients, composed of 254 (58.9%) men. Among the study participants, 194 expired in the ICU accounting for a case fatality rate (CFR) of 45%. CFR was found to be 42.1% (107) and 49.2% (87) among males and females, respectively, with no statistically significant difference [RR = 0.86 (0.70–1.06), *p* = 0.146]. Among the 416 patients, 291 (70%) were diagnosed using the rapid antigen test (RAT), followed by 111 (26.7%) by RTPCR and 14 (3.3%) by the nucleic acid amplification test (NAAT). The average time to diagnosis from the onset of symptoms was 3 days and that to MDICU admission was a week (Table 1). A delay in admission to the MDICU was significantly high among the deceased compared to survivors (*p* = 0.006).

**Table 1:** Natural history of events in days.

Variable	Mean ± SD	Median ± IQR	Minimum	Maximum
Starting of symptoms to diagnosis	3.37 ± 2.97	3 (1–4)	0	20
Starting of symptoms to MDICU admission	7.74 ± 5.53	7 (4–10)	0	35
Starting of symptoms to discharge /death	19.67 ± 9.84	18 (12–25)	1	64

Treatment was provided based on the Kerala State Medical Board protocol. Steroids were administered to all patients. Anticoagulants were provided in 301 (69.8%), Remdesivir in 274 (63.6%), Tocilizumab in 90 (20.9%), Baricitinib in 80 (18.6%), and Casirivimab-Imdevimab monoclonal antibody cocktail in 18 (4.2%).

### Predictors of mortality

#### Age

The CFR had an increasing trend with age from 25.7% in less than 40 years (reference category) to 73.3% in 70–79 years. The odds ratio (OR) for mortality was eight times in patients 70 years or above from the reference category (Chi-square for linear trend *p* value < 0.001). The age distribution and age-specific CFRs are given in Table 2. The CFR was 63.8% (67/105) for people with 60 years or more and 39% for those under 60 years (127/326) [RR = 2.76 (1.75–4.35), *p* < 0.001].

**Table 2:** Dose–response relationship of age, multimorbidity and biochemical parameters with probability of dying.

Variable	Categories	Frequency (percentage)	Case fatality rate	OR	Chi-square for linear trend *p* value
Age in years *N* = 431	<40	141 (32.7%)	36 (25.7%)	Ref = 1	<0.001
	40–49	92 (21.3%)	42 (45.7%)	2.45	
	50–59	85 (19.7%)	44 (51.8%)	3.13	
	60–69	68 (15.8%)	39 (57.4%)	3.92	
	70 and above	45 (10.4%)	33 (73.3%)	8.02	
Comorbidity *N* = 431	No comorbidity	111 (25.8%)	44 (39.6%)	Ref = 1	<0.001
	Single comorbidity	96 (22.3%)	31 (32.3%)	0.73	
	Multi-morbidity with two comorbidities	118 (27.4%)	53 (44.9%)	1.24	
	Multi-morbidity with three comorbidities	62 (14.4%)	34 (54.8%)	1.85	
	Multi-morbidity with more than three comorbidities	44 (10.2%)	31 (70.5%)	3.63	
Vaccination (*N* = 423)	Two doses	41 (9.7%)	10 (24.4%)	Ref = 1	0.002
	One dose	44 (10.4%)	16 (36.4%)	1.77	
	Unvaccinated	338 (79.9%)	164 (48.5%)	2.92	
Serum Ferritin at the time of admission (*N* = 361)	<500	154 (42.7%)	33 (21.4%)	Ref = 1	<0.001
	500–1,500	139 (38.5%)	58 (41.7%)	2.63	
	>1,500	68 (18.8%)	33 (48.5%)	3.46	
LDH at the time of admission (*N* = 341)	<280	18 (5.3%)	2 (11.1%)	Ref = 1	<0.001
	280–560	120 (35.2%)	24 (20%)	2.00	
	561–840	91 (26.7%)	29 (31.9%)	3.74	
	841–1,120	47 (13.8%)	24 (51.1%)	8.35	
	>1,120	65 (19.1%)	36 (55.4%)	9.93	
D-Dimer *N* = 353	5 or less	279 (79%)	107 (38.4%)	Ref = 1	<0.001
	5.1–10	54 (15.3%)	36 (66.6%)	3.2	
	>10	20 (5.7%)	13 (65%)	3.0	
SGOT at admission (*N* = 375)	<40	168 (44.8%)	51 (30.4)	Ref = 1	0.028
	40–120	169 (45.1%)	58 (34.3)	1.20	
	>120	38 (10.1%)	20 (52.6)	2.55	
SGPT at admission (*N* = 374)	<40	185 (49.5%)	60 (32.4)	Ref = 1	0.272
	40–120	155 (41.4%)	54 (34.8)	1.11	
	>120	34 (9.1%)	15 (44.1)	1.65	
Serum creatinine (*N* = 403)	<2	354 (87.8%)	143 (40.4%)	Ref = 1	<0.001
	2–5	24 (6%)	19 (79.2%)	5.61	
	>5	25 (6.2%)	16 (64%)	2.62	
Blood Urea (*N* = 400)	<40	220 (55%)	65 (29.5%)	Ref = 1	<0.001
	40–120	140 (35%)	77 (55%)	2.92	
	>120	40 (20%)	35 (87.5%)	16.69	

#### Clinical symptoms

A comparison of symptoms at the time of admission, between the deceased and survivors, showed that breathlessness (70.1% vs. 56.5%), cough (58.2% vs. 48.5%), fever (56.7% vs. 52.7%) and diarrhea (7.7% vs. 2.9%) were significantly more among the deceased (Table 3).

**Table 3:** Symptom profile, comorbidities, and complications among the deceased and survivors.

Symptom/comorbidity	Among expired *N* = 194 (100%)	Among survived *N* = 237 (100%)	RR (95%CI)	*p* value
Symptoms at the time of presentation				
Breathlessness	136 (70.1)	134 (56.5)	1.24 (1.07–1.43)	0.004
Cough	113 (58.2)	115 (48.5)	1.20 (1.01–1.43)	0.043
Fever	110 (56.7)	125 (52.7)	1.08 (0.91–1.28)	0.410
Diarrhea	15 (7.7)	7 (2.9)	2.62 (1.09–6.29)	0.032
Generalized weakness	14 (7.2)	13 (5.5)	1.32 (0.63–2.73)	0.736
Headache	15 (7.7)	25 (10.5)	0.73 (0.40–1.36)	0.319
Muscle pain	14 (7.2)	25 (10.5)	0.68 (0.58–3.10)	0.235
Throat congestion	11 (5.7)	10 (4.2)	1.34 (0.72–1.81)	0.488
Tiredness	9 (4.6)	13 (5.5)	0.85 (0.37–1.94)	0.692
Vomiting	13 (6.7)	19 (8.0)	0.84 (0.42–1.65)	0.605
Altered sensorium	6	3		
Decreased urine output	1	1		
Hemoptysis	0	4		
Nausea	4	6		
Known comorbidities at the time of admission				
Any form of hypersensitivity	6 (3.1)	12 (5.1)	0.82 (0.58–1.15)	0.309
Bronchial Asthma	11 (5.7)	14 (5.9)	0.98 (0.69–1.40)	0.917
Cerebro Vascular Accidents	8 (4.1)	4 (1.7)	1.67 (0.75–3.73)	0.126
Chronic Kidney Disease	17 (8.8)	8 (3.4)	1.76 (1.00–3.14)	0.017
Coronary Artery Disease	25 (12.9)	14 (5.9)	1.59 (1.03–2.43)	0.012
Chronic obstructive pulmonary disease	7 (3.6)	4 (1.7)	1.53 (0.70–3.35)	0.208
Diabetes mellitus	71 (36.6)	65 (27.4)	1.22 (1.00–1.49)	0.042
Dyslipidaemia	14 (7.2)	10 (4.2)	1.34 (0.83–2.17)	0.177
Hypertension	80 (41.2)	47 (19.8)	1.69 (1.32–2.15)	<0.001
Hypothyroidism	15 (7.7)	16 (6.8)	1.07 (0.75–1.52)	0.695
Malignancy	16 (8.2)	7 (3.0)	2.79 (1.17–6.65)	0.020
Obesity	4 (2.1)	6 (2.5)	0.91 (0.55–1.53)	0.747
Reactive airway disease	1	0		
Tuberculosis	1	2		
Psychiatric morbidities	5	7		
Chronic Liver Disease	2	2		
Complications during hospital stay				
COVID ARDS	35 (18.0)	8 (3.4)	5.34 (2.54–11.25)	<0.001
COVID Bronchopneumonia	148 (76.3)	156 (65.8)	1.16 (1.03–1.31)	0.017
Sepsis	21 (10.8)	6 (2.6)	4.28 (1.76–10.38)	0.001
Septic shock	21 (10.8)	1	25.65 (3.48–189)	0.002
A/c kidney injury	26 (13.4)	3 (1.3)	10.59 (3.25–34.45)	<0.001
A/c on Chronic kidney injury	3	0		
Infection related ventilator associated condition (IVAC)	54 (27.8)	31 (13.4)	2.13 (1.43–3.17)	<0.001
Catheter related blood stream infection	2	1		
Catheter associated UTI	1	0		
Hypothyroidism	17 (8.8)	11 (4.8)	1.89 (0.91–3.93)	0.09
Hyperthyroidism	0	2		
Acute coronary syndrome	2	3		
A/c liver failure	1	1		
Cardiogenic shock	3	1		
CVA hemorrhage	2	1		
CVA infarct	3	0		
Encephalitis	2	0		
Hepatitis	2	1		
Pleural effusion	1	3		
Pulmonary embolism	0	1		
Thrombocytopenia	4	3		

#### Comorbidities

At the time of admission, at least one known comorbidity was present in 74.2% (320/431) patients, with the highest proportion being diabetes mellitus, (136, 31.6%) followed by systemic arterial hypertension (127, 29.5%) (Table 3). However, 224 (70%) of the320 individuals with comorbidities suffered multi-morbidity which in turn was associated with a high CFR. The CFR had a dose–response relationship with the number of comorbidities. The odds of mortality in multi-morbidity with three or more existing diseases was 3.63 times compared to people without any reported comorbidities (Chi-square for linear trend *p* value < 0.001). Among individuals with comorbidities, those with chronic kidney disease [RR 1.7 (1.00–3.14), *p* = 0.022], systemic arterial hypertension [RR = 1.69 (1.32–2.15), *p* < 0.001], coronary artery disease [RR = 1.59 (1.03–2.43), *p* = 0.012], diabetes mellitus [RR = 1.22 (1.00–1.49), *p* = 0.042] and malignancy [RR = 2.79 (1.17–6.65), *p* = 0.020] were significantly associated with higher in-hospital mortality among critically ill patients.

#### Vaccination status

Among 423 critically ill patients who reported the vaccination status, 44 (10.4%) received a single vaccine dose and 41 (9.7%) received both doses. Among the vaccinated, 71 (84.5%) received the ChAdOx1 nCoV-19 Corona virus vaccine (Covishield) and 14 (15.5%) received the BBV152-inactivated vaccine (Covaxin). The CFR among unvaccinated was 48.5% (164/338), 36.4% (16/44) among those who received a single dose, and 24.4% (10/41) if both doses were received. People who took at least a single dose of vaccine was found to be significantly protected from mortality [RR = 0.63 (0.45–0.88), *p* = 0.008]. A dose–response relationship was noted between the number of vaccine shots and mortality. Compared to those who took two doses of vaccine, the odds ratio for mortality for those who received a single dose was 1.77, and in the unvaccinated group, it was 2.92 (Chi-square for linear trend *p* value = 0.002). There was no significant difference in mortality found among patients vaccinated with Covishield (29.6%) and Covaxin (35.7%), (*p* = 0.640).

#### Complications

Among the critically ill patients, 288 (66.8%) needed ventilatory support with, 248 (86.1%) receiving noninvasive ventilation. CFR was significantly more with COVID ARDS [RR = 5.34 (2.54–11.25)], COVID Bronchopneumonia [RR = 1.16 (1.03–1.31)], sepsis [RR = 4.28 (1.76–10.38)], septic shock [RR = 25.65 (3.48–189)], acute kidney injury [RR = 10.59 (3.25–34.45)] and infection-related ventilator-associated condition (IVAC) [RR = 2.13 (1.43–3.17)]. Most patients with bacteremia (72%) died; causative agents isolated include multi-drug resistant (MDR) acinetobacter, klebsiella, pseudomonas, and enterococci. Clinical complications observed during the study period are listed in Table 3.

#### Biochemical and radiological parameters

Serum ferritin, LDH, d-dimer, and SGOT were detected to have a dose–response gradient with the risk of mortality risk. The mortality risk increased with SGPT values, though it was not statistically significant (Table 2). NT-ProBNP values were more than 500 pg./mL in 33 of the 52 deceased compared to 42 among 90 survivors [RR = 1.36 (1.01–1.84) *p* = 0.046]. The PaO2/ FiO2 ratio was less than 150 in 58 of 83 deceased subjects compared to 53 among 156 survived [RR = 2.06 (1.59–2.67) *p* < 0.001]. A CRP of more than 100 mg/dL was observed in only 3 of the 113 expired patients compared to 2 among 226 surviving patients, the difference though not significant statistically. Chest skiagrams of patients showed evidence of bilateral infiltrates, peripheral opacities, consolidation of the lung parenchyma, pleural effusion, and cardiomegaly. Lung ultrasound showed poor aeration in 44.3% of survivors and 55.7% of non-survivors.

#### Principal components of mortality predictors

Nine domains/sets of variables that contributed to higher mortality were identified in the principal component analysis: (1) renal insufficiency, (2) hepatic insufficiency, (3) coronary artery disease and elevated inflammatory markers, (4) hypertension, diabetes, and lack of vaccination, (5) Pneumonia, (6) Breathlessness and ARDS, (7) sepsis and septic shock, (8) cough and (9) diarrhea at the time of admission. The variable “age more than 60 years” was found to be loaded with many domains (Table 4).

**Table 4:** Principal component analysis for grouping predictors of mortality (total score = 21).

Sl No	Principal component (number of subcomponents)	Variable	Factor loading
1	Renal (3)	Creatinine value more than 2	0.789
		Known case of renal failure	0.567
		Blood urea more than 40	0.651
2	Hepatic (3)	SGOT more than 40	0.824
		SGPT more than 40	0.646
		LDH more than 560	0.463
3	Cardiac and Inflammatory (4)	Known Coronary artery diseases	0.482
		D-dimer more than 5	0.747
		Ferritin more than 500	0.439
		LDH more than 560	0.440
4	Comorbidities and no-vaccination (3)	Hypertension	0.645
		Diabetes	0.713
		Status of no-vaccination	0.567
5	Pneumonia (2)	Respiratory Rate more than 30	0.720
		Clinical diagnosis of bronchopneumonia	0.744
6	Breathlessness and ARDS (2)	Breathlessness	0.759
		COVID ARDS	0.672
7	Sepsis (2)	Sepsis	0.707
		Septic shock	0.675
8	Cough (1)	Cough	0.812
9	Diarrhea (1)	Diarrhea	0.808

## Discussion

The study cohort exhibited an in-hospital mortality rate of 45%; nevertheless, it is imperative to contextualize this figure within the framework of individuals infected with SARS-CoV-2, who possessed the highest antecedent probability of mortality (patients studied fall into the highest category of clinical severity and were admitted in multidisciplinary ICU of a COVID hospital), as delineated within the study. However, the general community of Kerala experienced one of the lowest CFRs during the COVID-19 pandemic in India (5, 8).

Multiple studies have shown that age and comorbidities are associated with an increased risk of mortality and the relation is directly proportional, like the present study (9–11). The case fatality in COVID-19 grows exponentially with age. The relative immune deficiency from aging (immunosenescence), weakening of antiviral defenses and age-related comorbidities are factors that make age a crucial determinant of COVID-19-related mortality (12). The senior citizens are at increased risk of cytokine storm because of an increase in activity of NLRP-3 (nucleotide binding oligomerization domain) due to SARS-CoV-2 induced decline in sirtuin-2 levels (13). In the present study, 77.3% of the patients who died had comorbidities and CFR has established a dose–response relationship with the number of comorbidities. A study conducted in the UK by Docherty et al. (14) showed that heart disease, COPD, chronic kidney disease, obesity, and liver disorders are associated with a significant increase in COVID-19-related mortality. In a meta-analysis of 42 studies, comorbidities associated with increased mortality in COVID-19 were identified as chronic obstructive pulmonary disease (COPD), diabetes mellitus, systemic arterial hypertension, cerebrovascular disease, malignancy, and obesity (15). In our study patients who developed acute kidney injury (AKI) ARDS, sepsis, septic shock, and IVAC had a significantly increased risk of death. Bacterial superinfections due to MDR pathogens are a significant cause of mortality in severe COVID-19 cases. Critically ill require invasive device insertions and receive immunomodulators like steroids, tocilizumab, and JAK inhibitors which make them susceptible to the development of healthcare-associated infections due to MDR pathogens (16). Most of the bacterial superinfections in COVID-19 are healthcare-associated and a recent study showed that unsuccessfully treated ventilator-associated bacterial pneumonia in patients with severe COVID-19 is associated with increased risk of mortality risk (17, 18).

In the present study, the mortality was significantly lower among patients who had received SARS-CoV-2 vaccination. A dose–response relationship was noted between the number of vaccine shots and mortality. Our study group is not a good cohort to assess the vaccine effectiveness because vaccines would have reduced the probability of people entering into the category of severe COVID-19 disease. Nevertheless, this study provides evidence that even among critically ill patients, vaccination is protective. Studies have shown that full vaccination status had a mortality benefit even in patients who required mechanical ventilation due to COVID-19-related ARDS (19, 20). A large study by Baker et al. (20) in hospitalized adult patients with COVID-19 showed that vaccination was associated with significant reductions in mortality for obese, severely obese, and older adult patients. Vaccine effectiveness studies have proved that 2 or 3 doses of vaccine can result in a 90% reduction in risk for severe COVID-19 outcomes, including invasive mechanical ventilation and in-hospital death across all variant periods (21, 22). Studies have shown that hybrid immunity is superior to vaccine or infection-induced immunity in preventing COVID-19-related adverse clinical outcomes (23).

Several biochemical parameters were also significantly associated with mortality in the present study. Serum ferritin is an acute phase reactant whose levels reflect the hyperinflammatory milieu prevailing in the body due to COVID-19 and could be used as a predictive biomarker for assessing COVID-19 severity (24). Elevated LDH, SGOT, and SGPT are reflections of cellular hypoxia attributed to the reduced PaO2/FiO2 occurring in severe COVID-19 pneumonia. Transaminitis can be due to liver injury by the virus, proinflammatory cytokines, congestive hepatopathy, hypoxia, ischemia, drugs, or due to myositis. Elevated LDH level mirrors the extent of cell membrane necrosis and increasing level of LDH correlates with the extent of tissue damage and inflammation. In the current study, Lung ultrasound (LUS) showed poor aeration in 44.3% of survivors and 55.7% of non-survivors. This difference was statistically significant. In contrast to other studies, in our study, the CT severity index (CTSI) > 15 was not found to be a predictor of mortality. This shows that for functional evaluation of lung aeration in COVID-19 pneumonia, LUS is superior to CTSI. A high LUS score is associated with unfavorable outcomes. Multiple studies have shown that LUS helps diagnose, prognosticate, and initiate optimal critical care interventions without delay. The study has also identified nine domains that contributed to the mortality. Multiple studies have shown that in SARS-CoV-2 patients with cardiovascular disease, elevated markers of thrombo-inflammatory activation like CRP, IL-6, and troponins are predictors of mortality (25). The study points out the importance of infection control practices in ICUs to reduce mortality among critically ill patients. It highlights the need for caregivers to be adequately trained and consistently vigilant in mitigating hospital acquired infections.

**Figure 1:**
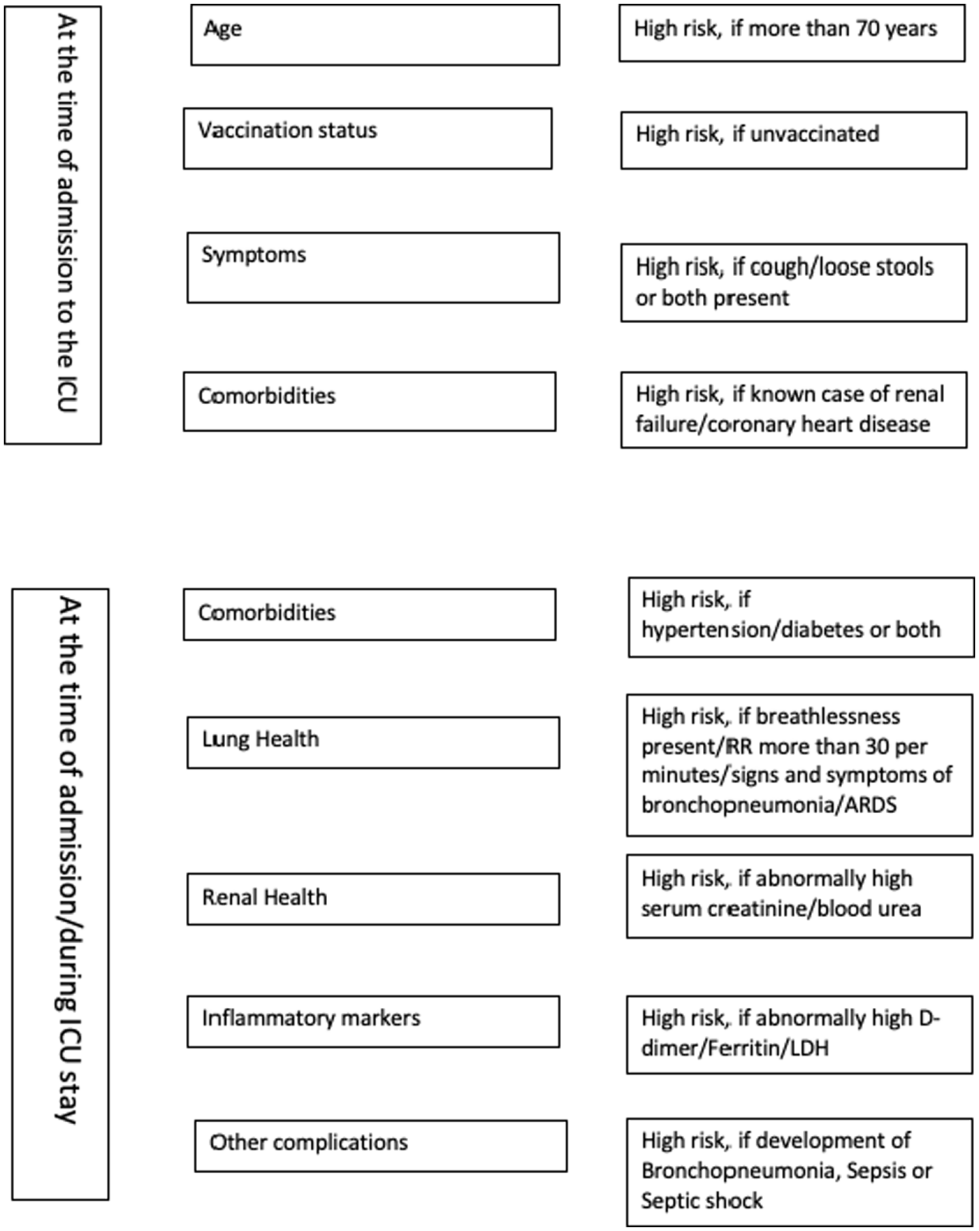
Risk stratification checklist for critically ill COVID-19 patients admitted in ICUs.

We used principal component analysis to identify the syndrome complexes and correlated factors at the time of admissions, those contributed to mortality and identified nine domains. It included renal insufficiency, transaminitis, coronary artery disease and elevated inflammatory markers, comorbidities and being unvaccinated, pneumonia, breathlessness and ARDS, sepsis and septic shock, cough/diarrhea at the time of admission. We suggest a 21-item scoring system to assess the mortality risk for critically ill COVID-19 patients admitted in ICUs (Table 4). The minimum score is 0 and maximum score 21 and the risk of mortality increases with the score. Old age may be included as another component at the time of validation as age was found to be loaded in multiple principal components. The checklist given as Figure 1 could be used by the emergency physician for the risk stratification of critically ill COVID-19 patients admitted in ICUs.

### Limitations of the study

Being a retrospective study, the completeness of data to certain variables was a major limitation. The current study was conducted in a single hospital in Kerala and it may not be a representative case of the pan Indian scenario. We presented the distribution of patients who received steroids, antiviral drugs and anticoagulants. However, these treatments were administered based on specific clinical indications, and to those patients with higher risk of in-hospital mortality. The potential selection bias prevented us from performing a specific analysis on the impact of treatments. This study focused solely on in-hospital mortality and did not analyze the mortality risk and contributing factors over a longer time frame. The tool that we proposed to measure the in hospital mortality of critically ill in COVID-19 needs further validation, to assign weightages for different subcomponents, and to find out the cutoff point to predict in-hospital mortality.

## Conclusion

In low-and -middle-income countries with limited health system resources, the nine domains identified as main predictors of mortality among critically ill patients in this study will aid the practitioners in triaging the patients and in improving the clinical outcomes. The identified predictors help in prognosticating patient outcomes and thereby guide the rationalization of clinical management. The findings of the current study can help inform the prioritization of patients admitted to intensive care units in hospitals located in LMICs during pandemics and large-scale outbreaks caused by respiratory pathogens.

## Data Availability

The raw data supporting the conclusions of this article will be made available by the authors, without undue reservation on request to the corresponding author.
